# A Direct Relationship Between ‘Blood Stasis’ and Fibrinaloid Microclots in Chronic, Inflammatory, and Vascular Diseases, and Some Traditional Natural Products Approaches to Treatment

**DOI:** 10.3390/ph18050712

**Published:** 2025-05-12

**Authors:** Douglas B. Kell, Etheresia Pretorius, Huihui Zhao

**Affiliations:** 1 Department of Biochemistry, Cell and Systems Biology, Institute of Systems, Molecular and Integrative Biology, Faculty of Health and Life Sciences, University of Liverpool, Crown St., Liverpool L69 7ZB, UK; 2 The Novo Nordisk Foundation Centre for Biosustainability, Technical University of Denmark, Søltofts Plads 200, 2800 Kongens Lyngby, Denmark; 3 Department of Physiological Sciences, Faculty of Science, Stellenbosch University, Stellenbosch Private Bag X1, Matieland 7602, South Africa; 4 School of Traditional Chinese Medicine, Beijing University of Chinese Medicine, Beijing 100026, China; hh686@126.com; 5 Institute of Ethnic Medicine and Pharmacy, Beijing University of Chinese Medicine, Beijing 100026, China

**Keywords:** blood stasis, clotting, amyloid, fibrinaloid, natural products, bioactive molecules, Chinese Herbal Medicine, inflammation

## Abstract

‘Blood stasis’ (syndrome) (BSS) is a fundamental concept in Traditional Chinese Medicine (TCM), where it is known as Xue Yu (血瘀). Similar concepts exist in Traditional Korean Medicine (‘Eohyul’) and in Japanese Kampo medicine (Oketsu). Blood stasis is considered to underpin a large variety of inflammatory diseases, though an exact equivalent in Western systems medicine is yet to be described. Some time ago we discovered that blood can clot into an anomalous amyloid form, creating what we have referred to as fibrinaloid microclots. These microclots occur in a great many chronic, inflammatory diseases are comparatively resistant to fibrinolysis, and thus have the ability to block microcapillaries and hence lower oxygen transfer to tissues, with multiple pathological consequences. We here develop the idea that it is precisely the fibrinaloid microclots that relate to, and are largely mechanistically responsible for, the traditional concept of blood stasis (a term also used by Virchow). First, the diseases known to be associated with microclots are all associated with blood stasis. Secondly, by blocking red blood cell transport, fibrinaloid microclots provide a simple mechanistic explanation for the physical slowing down (‘stasis’) of blood flow. Thirdly, Chinese herbal medicine formulae proposed to treat these diseases, especially Xue Fu Zhu Yu and its derivatives, are known mechanistically to be anticoagulatory and anti-inflammatory, consistent with the idea that they are actually helping to lower the levels of fibrinaloid microclots, plausibly in part by blocking catalysis of the polymerization of fibrinogen into an amyloid form. We rehearse some of the known actions of the constituent herbs of Xue Fu Zhu Yu and specific bioactive molecules that they contain. Consequently, such herbal formulations (and some of their components), which are comparatively little known to Western science and medicine, would seem to offer the opportunity to provide novel, safe, and useful treatments for chronic inflammatory diseases that display fibrinaloid microclots, including Myalgic Encephalopathy/Chronic Fatigue Syndrome, long COVID, and even ischemic stroke.

## 1. Introduction

### 1.1. Preamble, Audience, and Scope

This review integrates concepts of systems medicine from both Eastern and Western traditions. It is intended to be read (and readable) by scientists, clinicians, and patients from very different intellectual and cultural backgrounds, and will necessarily include subsections that are completely familiar to some but entirely arcane to others. We hope that at the end readers will recognize that the discovery of fibrinaloid microclots provides a ready general explanation for the traditional, if molecularly ill-defined, concept of ‘blood stasis’. Equivalently, the concepts (and extensive traditional knowledge) of blood stasis may provide extremely useful insights into the biology of fibrinaloid microclots and the means of treating them and the diseases with which they are associated. Our focus is on Chinese Herbal Medicine, and we acknowledge (but for reasons of scope we mainly do not rehearse) the underlying principles of Traditional Chinese Medicine (TCM), such as five-element theory and the analysis of pulses, meridians, and Qi [1]. We do not directly consider other adjunctive treatment elements of TCM such as acupuncture, moxibustion, cupping, massage, and so on, since the focus is the mode of action of relevant Chinese herbal formulae and the molecules they contain, as they relate to blood stasis. We then focus on a particular herbal formulation used to treat blood stasis and the nature and mechanisms of the bioactive chemicals that it contains. For reasons of accessibility we have avoided Chinese-language publications. A preprint has been lodged [2].

### 1.2. A Note on Systems and Personalized Medicine

Modern Western medicine, especially that based on pharmaceuticals, has tended to give the impression that a particular drug may be targeted at, and will be efficacious in, all patients. Given the existence of some 25,000 genes, each with many alleles, leav aside phenotypic variations such as those based on lifestyle effects, it has always been obvious that this could not be the case (e.g., [3]). As phrased by the 18th century physician Caleb Parry (quoted in [4]), ‘[i]t is much more important to know what kind of patient has a disease than to know what kind of disease a patient has’. This principle of personalized medicine (e.g., [5,6], including the role of AI therein [7,8]) lies at the heart of TCM and is intimately linked with equivalent Western concepts such as systems biology [9,10,11,12,13,14,15], systems medicine [16,17], polypharmacology [18,19,20,21,22,23,24,25,26,27,28,29,30], and network pharmacology [31,32,33,34,35,36]. An early approach to this (that could be seen as a subset of systems biology) known as metabolic control analysis (e.g., [37,38,39,40,41,42,43,44]) describes explicitly how and why individual biochemical reactions contribute only weakly to the control of metabolic fluxes, and why, to have big effects, one must modulate multiple reactions simultaneously. Natural evolution selects for robustness to individual insults [45], and the kinetic and architectural [46] properties of such networks tend to provide it; indeed, the interlinked kinetics of biochemical networks mean that it is easy to find circumstances in which two inhibitors individually have negligible effects on a metabolic flux, whereas together their effect can be massive [47,48].

As a systems approach, Chinese herbal medicine adheres to the **Jun-Chen-Zuo-Shi principle** [49,50,51,52,53] (Westerners will find some minor variations of the Chinese characters). As phrased by [50], ‘[t]he Jun (emperor) component is the principal phytocomplex targeting the major symptom of the disease. There are only a few varieties of Jun medicinals that are administered as a single formula, usually in large doses. The Chen (minister) herbs synergize with Jun to strengthen its therapeutic effects, and may also treat secondary symptoms. The Zuo (assistant) medicinal reduces or eliminates possible adverse or toxic effects of the Jun and/or Chen components, while also enhancing their effects and sometimes treating secondary symptoms. Finally, the Shi (courier) herbs facilitate delivery of the principal components to the lesion sites, or facilitate the overall action of the other components’. It is particularly interesting that the last component effectively relates to the significance of pharmaceutical drug transporter proteins, something finally being recognized more widely (e.g., [54,55,56,57,58]) and that in fact had evolved precisely to transport natural products [59].

While recognizing that TCM practitioners will vary treatments precisely to suit the individual, we do not normally have access to such information for our scientific purposes. Equally, we rarely know the multiple targets of even single pharmaceutical drugs [60] (in 2008 the average number of known targets per drug molecule was six [61]). Consequently, this review will seek to paint a big picture, recognizing in particular that it is combinations of herbs affecting multiple processes that have the greatest chance of having useful effects [34,62,63,64,65,66], while seeking to bring together the biochemistry of microclots (see below) with what is known of blood stasis.

### 1.3. Blood Stasis

‘Blood stasis’ (or blood stasis syndrome, BSS) is a fundamental concept in Traditional Chinese Medicine (TCM), where it is known as Xue Yu (血瘀) [67]. BSS refers to conditions in which the circulation of blood is not smooth or is slowed down in some way (e.g., [68,69], and see later). It has been known (using other terms) at least from the time of *The Yellow Emperor’s Inner Classic* (Huang Di Nei Jing) [67,70]. The same concept exists in many other traditional medicines, including Traditional Korean Medicine (where blood stasis is known as ‘Eohyul’ or ‘Ouhyul’) [15,71] and in Japanese Kampo medicine (where it is termed Oketsu). Even within TCM there are similar variants (e.g., [72]). Overall, while we recognize that there are nuanced differences of interpretation both within and between these traditions, and probably also among individual practitioners, it is also recognized that blood stasis may in fact manifest differently in different populations [73,74], and this cannot easily be deconvolved. Blood stasis can be caused by vascular obstruction, abnormal flow of blood, and blood congestion in viscous, contaminated blood, and has at least four subtypes [75]. It is regarded as the cause or the result of a great many chronic inflammatory diseases [76,77,78], which we summarize later. Many general reviews of blood stasis exist, e.g., [68,73,76,79,80,81,82,83].

Traditionally, BSS is measured somewhat subjectively by a practitioner’s observation of symptoms or manifestations such as tongue color and the results of palpations. More recently, attempts have been made to objectify or quantify the extent of BSS using various kinds of scores (e.g., [70,84,85,86,87,88,89,90,91,92,93,94,95]). Blood stasis is fundamentally related to hemorheology measurements (i.e., viscosity) [88,96,97]. We rehearse explicitly this point about scoring BSS, as such quantitative measurements (as used, e.g., for scoring fatigue in long COVID [98]) will be highly desirable for future studies that seek to relate microclot burden to BSS. However, such data presently do not exist.

It is also worth rehearsing here that the 19th-century physiologist Virchow recognized three factors (known as Virchow’s triad [99,100,101,102,103]) that contribute to the development of venous thrombosis, and these are stasis of blood flow, hypercoagulability, and endothelial injury. The significance of the terminology of the first one is not lost on us, and the parallel between ‘stasis of blood flow’ in Virchow’s triad and BSS supports a potential physiological basis for this traditional concept. However, the precise overlap of such terminology with biomedical phenomena and terminology more commonly used nowadays in Western medicine, such as vascular insufficiency, microcirculation disorders, or coagulopathies, is not at all established, and, as mentioned [73,74], the manifestation of blood stasis syndrome may even differ between populations. To this end, part of the purpose of this review is to suggest that it would be very desirable to address these issues.

### 1.4. Fibrinaloid Microclots

**Figure 1:**
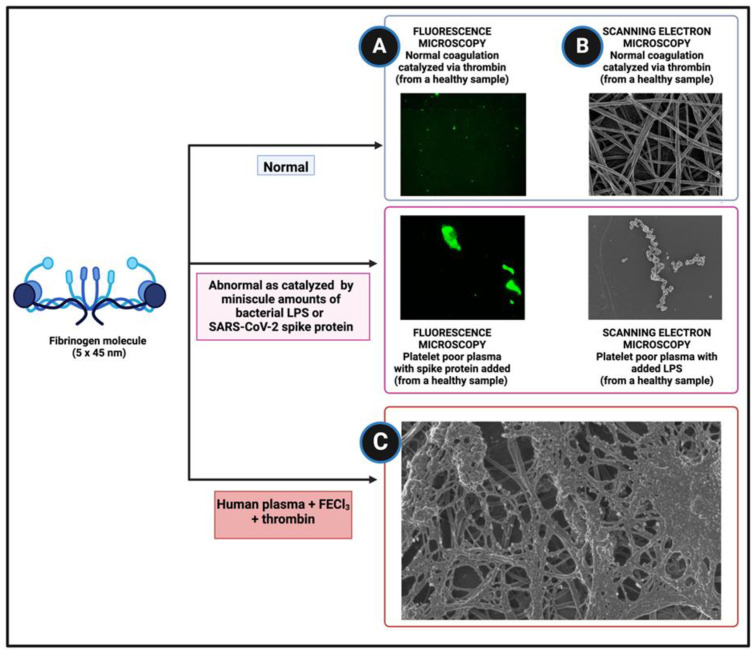
Structure and dimensions of fibrinogen and its folding into healthy and pathological amyloid fibrin(ogen). (**A**) Fluorescence microscopy of healthy plasma (with and without spike protein), with thioflavin T (to show amyloid areas in fibrin(ogen)) and added thrombin. (**B**) Scanning electron microscopy of fibrin(ogen) with and without lipopolysaccharide (LPS) and thrombin (**C**) Scanning electron microscopy of human plasma with FeCl_3_ and thrombin. (Adapted from [111,141]). Generated with Biorender.com.

Fibrinogen, one of the most abundant plasma proteins (2–4 g·L^−1^), has dimensions of some 5 × 45 nm, giving a length:diameter ratio of ~9. As is well known, a key part of blood clotting involves the removal from fibrinogen by the protease thrombin of two fibrinopeptides (FpA and FpB), leading to a remarkable self-organization in which fibrinogen molecules accrete to form far larger fibrils and protofibrils via a ‘knobs and stalks’ mechanism (Figure 1A–C). In normal clotting, the fibrinogen molecules are essentially oriented in the direction of the growing chain, which looks somewhat like cooked spaghetti in the electron microscope (EM) (Figure 1B). Other things such as erythrocytes and platelets may also be trapped, along with non-fibrin proteins whose concentrations in the clot roughly correlate with those in normal soluble plasma [104,105].

Over a decade ago, it was discovered was that certain small molecules such as specific estrogens [106,107] or unliganded iron [108,109,110,111,112,113] could cause fibrinogen to form highly anomalous clots that in the EM appeared like claggy aggregations of partly boiled spaghetti, and that were referred to at the time as ‘dense matted deposits’ [110,111,114,115,116] (Figure 1C). Their properties differ from those of normal clots in a variety of different ways, and at the helpful suggestion of a referee, Table 1 summarizes the main differences we have noticed between ‘conventional’ clots and the fibrinaloid (micro)clots that we discovered.

**Table 1:** Some differences between the properties of fibrinaloid microclots and those of normal blood clots (see also [117]).

Property	Normal Clots	Fibrinaloid Microclots	Selected References
Appearance under an electron microscope	Like spaghetti cooked *al dente*	Like parboiled spaghetti congealed in a claggy mess (and originally known as ‘dense matted deposits’)	Figure 1 and [111,112,113,116,118,119,120,121]
Significant autofluorescence when excited at 405 nm or below (for one-photon excitation [122])	No	Yes (as with all amyloid proteins)	[123,124,125,126,127,128]
Fluoresce strongly when stained with thioflavin T or Amytracker dyes	No	Yes	[129,130]
High α-helix content	Yes	No	[120,131,132]
High β-sheet content	No	Yes	[120,131,132]
Lysis/fibrinolysis by various common proteases is relatively easy	Yes	No	[133,134]
Proteome of clot reflects plasma proteome	Yes	No	[131,132]
Proteome of clot rich in amyloidogenic proteins	No	Yes	[131,132]
Typical fiber diameter	Variable but ca 100 nm	Can be much greater	[129,131,135,136,137,138,139,140]

**Figure 2:**
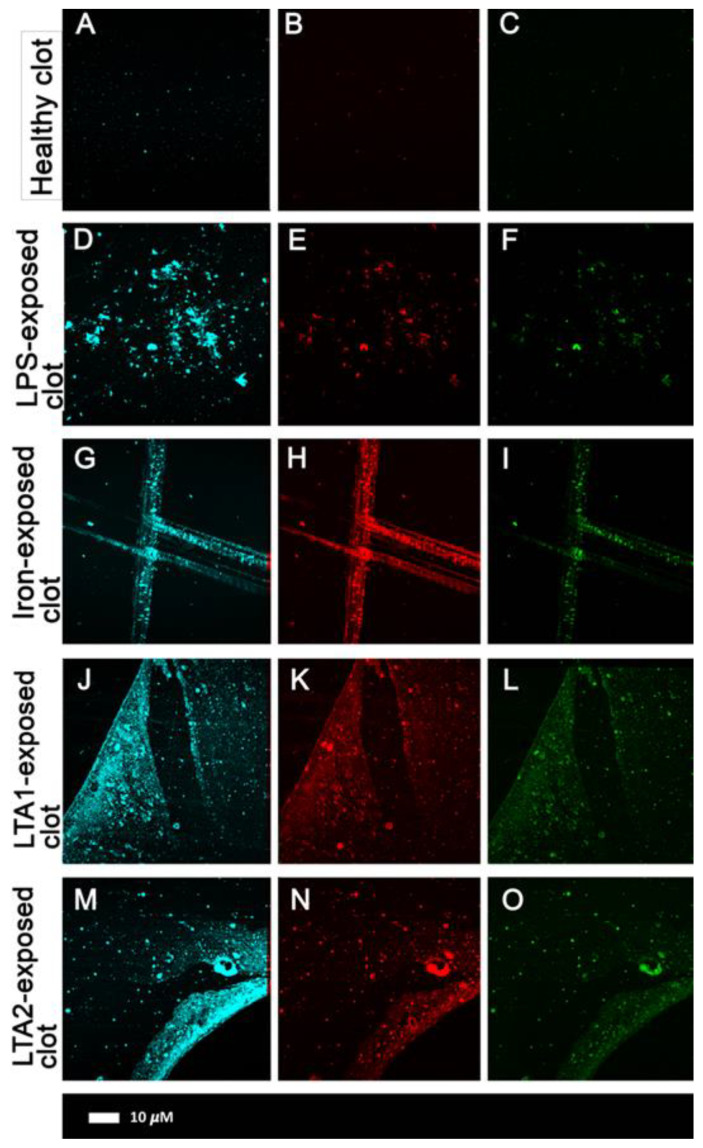
Representative confocal images of human plasma with three amyloid markers (cyan: Amytracker™ 480; red: Amytracker™ 680; green: ThT). (**A**–**C**) Naïve human plasma; (**D**–**F**) plasma exposed to lipopolysaccharide (LPS); (**G**–**I**) plasma exposed to iron; (**J**–**L**) plasma exposed to lipoteichoic acid-1; (**M**–**O**) plasma exposed to lipoteichoic acid-1. (Taken from a CC-BY Open Access publication [130]).

Subsequent studies, using the well-established [142,143,144] amyloid stain thioflavin T (Figure 2), as well as the commercial oligothiophene ‘Amytracker™’ stains [129,145], showed that this anomalous clotting (i) was actually due to conversion of the fibrin(ogen) into amyloid forms, which are characterized by cross-β motifs that bind these stains and effect their observable fluorescence, (ii) could be induced by minuscule amounts of bacterial cell wall components (e.g., one molecule of bacterial lipopolysaccharide per 100 million fibrinogen molecules [129]), and (iii) could be observed in a large variety of chronic, inflammatory diseases (e.g., [146] and Table 1). Much as with prions and other amyloid proteins [120,147], there is no thermodynamic problem; the clotting will happen anyway, and these molecules simply catalyze a different route of self-organization that maintains the necessary close packing in the relevant macrostates.

Clearly, such insoluble microclots (like other microparticulates [148,149]) can straightforwardly interfere with the flow of blood cells through microcapillaries, i.e., block them, leading to a loss of O_2_ transfer, hypoxia, and other pathological consequences [150]. Finally, here, we recognize that amyloid proteins generally share cross-β structural motifs that serve to bind stains such as thioflavin T [142,144,151,152,153,154,155,156,157,158,159,160,161], as well as ‘pan-amyloid’ peptides and antibodies (e.g., [162,163,164,165,166]).

## 2. Diseases Involving Blood Stasis in Which Raised Levels of Fibrinaloid Microclots Have Been Detected

Over the years, we and others have assessed the raised presence of fibrinaloid microclots in a series of chronic, inflammatory diseases, each of which, it transpires, is considered to involve blood stasis. Table 2 provides a summary. Note, of course, that many of these syndromes, especially those (as here) involving vascular issues, exhibit comorbidity with each other because they have common causes. Comorbid diabetes and Alzheimer’s disease provides one such example [146,167,168,169,170,171,172,173,174,175,176,177,178,179,180,181] of many. TCM of course recognizes this explicitly, where it is known as ‘Treating Different Diseases with the Same Treatment’ [83,182,183,184].

We would stress that each of these diseases is closely related to blood stasis syndrome. Thus, blood stasis syndrome is a commonality of these diseases. It is also a good bridge for communication between Chinese and Western medicine.

## 3. Diseases Involving Blood Stasis Where Fibrinaloid Microclots Are Yet to Be Measured Directly

**Figure 3:**
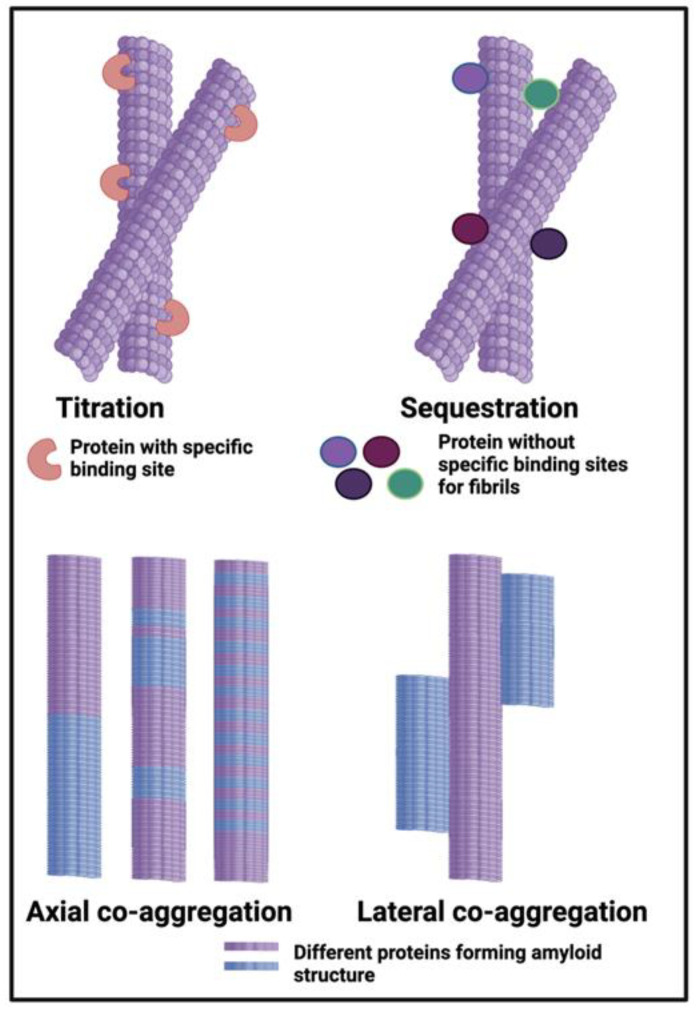
Weak binding or sequestration. Different classes or types of protein co-aggregation: titration; sequestration; axial and lateral. (Figure adapted from open access papers [131,132], based on [299]). Generated with Biorender.com.

In a similar vein, effectively the converse of the above, there are many diseases involving blood stasis in which microclots have yet to be assessed, but which would make obvious objects of study from this point of view. The basic reasoning is as per the paired papers [131,132] on amyloid clot proteomics. In the first [131], we showed that known amyloid microclots had proteomes that differed markedly from those of known (‘normal’) clots. Besides fibrin, they contained various proteins that were in low concentration in soluble plasma yet lacked many that were in high concentration there. Indeed, normal clots had a proteome that roughly mirrored the soluble plasma proteome, implying relatively weak binding or sequestration (see Figure 3). The proteins ‘enriched’ in the microclots were highly amyloidogenic, suggesting that they were actually incorporated into the fibrils via the cross-β motifs common to all amyloids. The second paper [132] asked the ‘inverse’ question, i.e., if we know the composition of the clot proteome in various thrombotic diseases, can we predict whether or not the clot is amyloid (ogenic)? In all cases, the answer was that these clots should indeed be amyloid, which can thus be tested (and in the case of ischemic stroke had been [277]).

Table 3 gives a listing of various vascular and thrombotic diseases that are known from TCM to be associated with blood stasis but that are not in Table 2, along with some mechanistic comments that suggest that studies assessing whether or not the microclots were both present and amyloid in character in these diseases would likely be attended with success.

**Figure 4:**
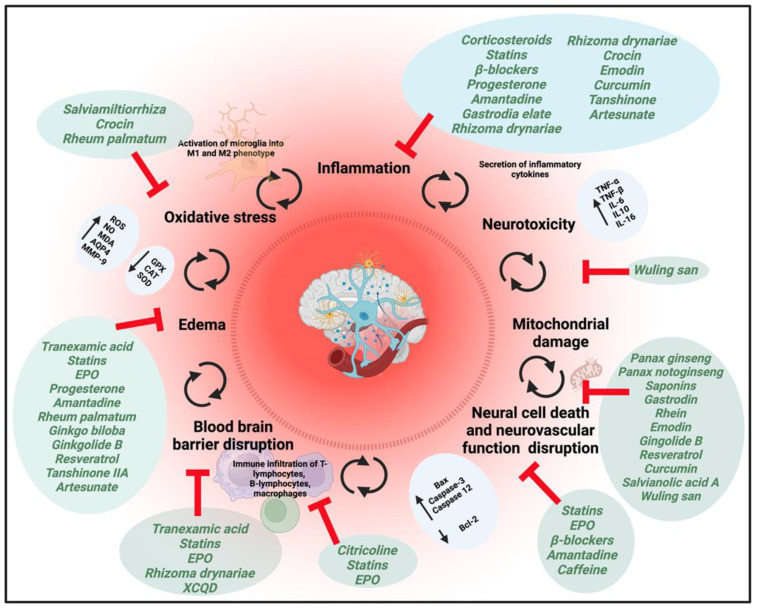
Multipotential drug treatment strategies for traumatic brain injury. Redrawn from [403]. Generated with Biorender.com.

**Figure 5:**
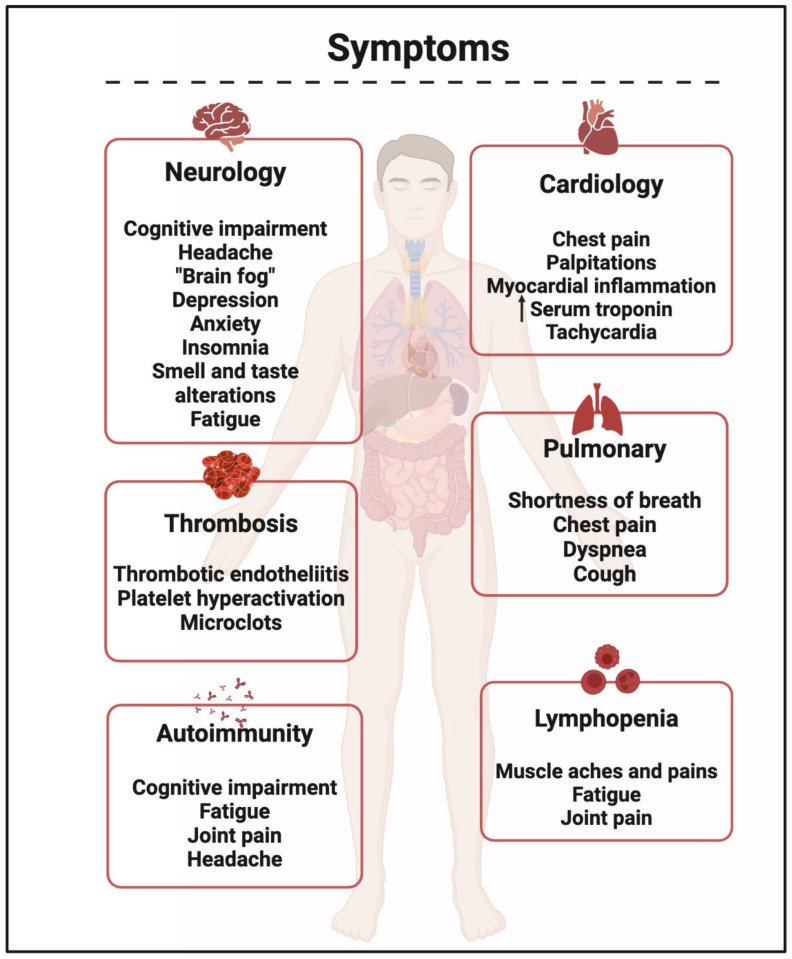
Long COVID symptoms (taken from [213]). Generated with Biorender.com.

These all tend to be systems diseases, and so the different components of herbal preparations will tend to interrogate different elements of what has been disrupted. An example from traumatic brain injury [403] is given in Figure 4, and one from long COVID, showing the multiplicity of symptoms, in Figure 5.

### Cancer and Classical Amyloidoses

We did not include the classical amyloidoses in the above table (though Alzheimer’s and Parkinson’s, listed in Table 2, would certainly fall into those categories), not least because there are a great variety of them (including polymorphs) [406,407,408,409,410,411,412,413,414,415,416], cross-seeding is commonplace (e.g., [131,417]), and they deserve a separate treatment in their own right. Similarly, cancer is a topic that is so broad and massive that it too deserves (and will receive) a separate treatment. Consequently, we here note only four things:

- Cancer, particularly pancreatic cancer (PDAC), is strongly linked to thromboembolic states [418,419,420,421,422,423,424,425,426,427,428,429,430,431,432,433,434,435], with thrombotic events often leading to a worse outcome.
- Cancer is strongly associated (as, of course, are long COVID [436,437,438,439,440,441,442,443] and ME/CFS [221,440,442,444,445]) with fatigue [446,447,448,449,450,451,452,453].
- Unsurprisingly, therefore, cancers are strongly associated with blood stasis [69,72,80,340,454,455,456,457,458,459,460,461].
- Consequently, components of Xue Fu Zhu Yu [462] such as saikosaponins (e.g., [463,464,465,466,467,468,469,470,471,472,473,474,475]) have been found to have efficacy as anti-cancer agents.

## 4. Amyloid Nature of the Blood Clots in Blood Stasis

While we are not aware of any measurement of the amyloid nature (or otherwise) of microclots in samples characterized by CHM practitioners as involving blood stasis, proteomics can on its own predict whether a clot is likely to be normal or amyloid in character [131,476], and all the recent assessments of the microclots occurring in these various diseases show that they are amyloid in character. Such proteins (that include prions and prionoids) are well known, because of the cross-β sheet motifs, to be rather resistant to most proteases [477,478,479,480,481,482,483]. This, together with the presence of various anti-fibrinolytics trapped in such clots [133,134], provides a ready explanation for the failure to remove them, such that they can contribute strongly to the phenomena of blood stasis.

## 5. A Focus on Xue Fu Zhu Yu

Having established the consonance between cases (accompanied by inflammation and coagulopathies) where fibrinaloid microclots have been measured and the co-existence of blood stasis as defined within TCM, it is of interest to begin to understand what these various herbs may be doing. As mentioned, even single pharmaceutical drugs have multiple known targets [61]; in some cases (such as statins, reviewed in [484]), the so-called ‘off-target’ effects are actually largely responsible for the efficacy of the drug in terms of increasing longevity. Consequently, deconvolving accurately what everything is doing within a Chinese herbal medicine cocktail is going to be very difficult. However, this does not mean that some progress cannot be made in terms of establishing components that, e.g., are anti-inflammatory or anti-oxidant and [336] provides a nice example for pulmonary fibrosis. To rehearse again, it is by hitting these multiple targets simultaneously that one can expect and find that the formulae are efficacious.

**Xue Fu Zhu Yu** (sometimes written as XueFu ZhuYu or Xuefuzhuyu) is an herb combination designed to boost Qi and remove blood stasis [485,486,487,488,489,490]. Xue Fu Zhu Yu decoction (Xue Fu Zhu Yu tang, XFZYD) is used explicitly for a variety of **coronary diseases** [302,307,309,310,341,343,352,491,492,493,494,495,496,497,498,499,500,501,502,503,504,505,506,507] (notably decreased mortality from ischemic heart disease by more than four-fold [508]), as well as **traumatic brain injury** [51,401,509,510,511,512,513,514,515,516,517,518,519,520], **NAFLD** [371,521] (nowadays known as **MASLD** [370]), **deep vein thrombosis** [356], **fibrosis** [522], **ischemic stroke** [504,523,524], **COPD** [335], **sepsis** [237] (including a five-herb injectable variant known as Xuebijing (XBJ, see below) [238,239,240,241,242,243,244,245,246,247,248,249,250,251,252,253,254,255,256,257,258,259,260,261,262,263]), **amyloidogenesis** [189], **myocardial fibrosis** [494], **dysmenorrhea** [357,359,360], **hypertension** [525], and **tumors** [462].

**Figure 6:**
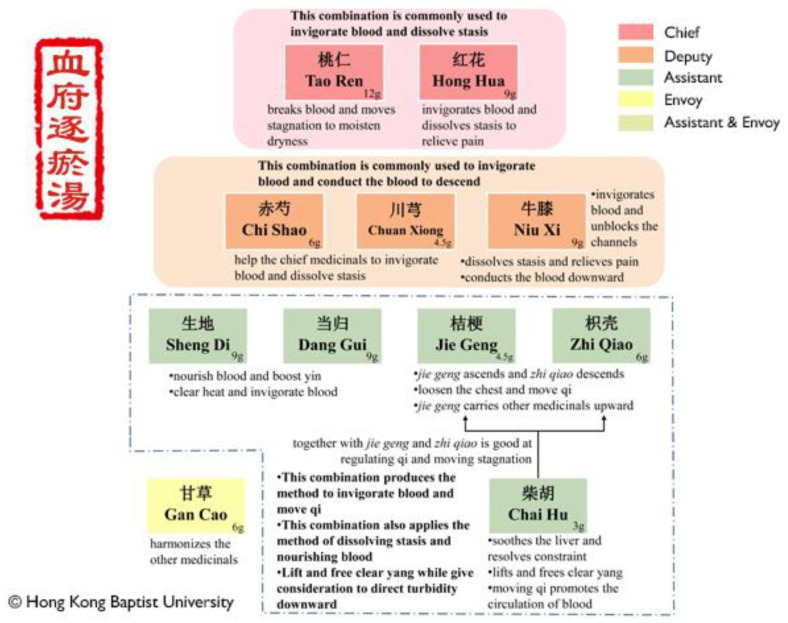
‘Blood stasis–expelling decoction’ (Xue Fu Zhu Yu tang) or ‘stasis in the mansion of blood’, showing (with the database owner’s permission) part of the page at https://sys02.lib.hkbu.edu.hk/cmfid/details.asp?lang=eng&id=F00115 (accessed on 6 May 2025).

**Figure 7:**
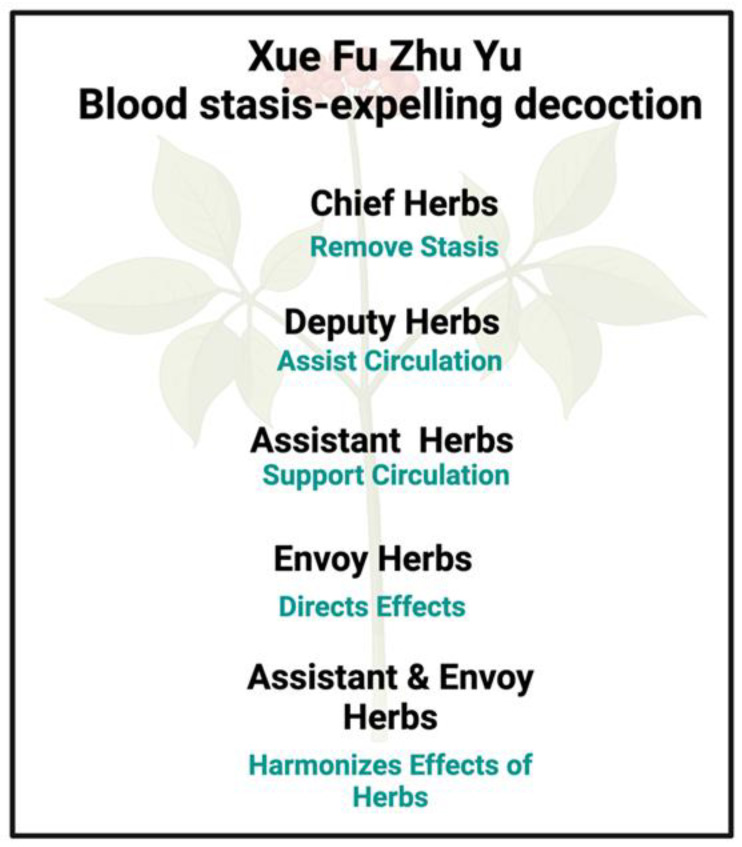
A summary of Xue Fu Zhu Yu, based on the material at https://sys02.lib.hkbu.edu.hk/cmfid/details.asp?lang=eng&id=F00115 (accessed on 6 May 2025).

For illustrative purposes, we are therefore going to concentrate on Xue Fu Zhu Yu (血府逐瘀); ‘blood stasis–expelling decoction’ or ‘stasis in the mansion of blood’), since, while others such as Danshen–Chuanxiong are certainly in use against some diseases of blood stasis (e.g., [526,527,528]), this Xue Fu Zhu Yu formula https://sys02.lib.hkbu.edu.hk/cmfid/details.asp?lang=eng&id=F00115 (accessed on 6 February 2025) is among those most commonly used to treat blood stasis (e.g., [341,487,490,497,529,530]). Figure 6 provides a screen dump from part of that page, while Figure 7 provides a summary analysis.

The aim here is to indicate the kind of knowledge we presently have of the most significant chemical components in each herb, while recognizing that some modest variations would likely be prescribed for individuals who are physically seen by a Chinese Medical Herbalist.

Xue Fu Zhu Yu, or Xuefu Zhuyu, has 11 herbal components [531,532,533]. Proportions vary, but we give the percentages in a particular preparation of which we are aware. The ingredients are *Semen persicae* aka *Prunus persica* = peach seed (Tao Ren) 16%, *Radix rehmanniae* from *Rehmannia glutinosa* Libosch (Di Huang or Sheng Di, depending on whether dried or fresh) 12%, *Radix achyranthis bidentata* or *Cyathulae radix* (Niu Xi) 12%, *Radix angelicae sinensis* (Chinese angelica) (Dang Gui) 12%, *Flos carthami* aka safflower (Hong Hua) 12%, *Fructus aurantia* (Zhi Qiao) 8%, *Radix paeoniae rubra* (Chi Shao) 8%, *Radix platycodonis* (Jie Geng) 6%, *Rhizoma chuanxiong* (*Ligusticum chuanxiong*) or Szechaun lovage roots 6%, *Radix glycorrhizae* (Chinese licorice) (Gan Cao) 4%, and *Radix bupleuri* (Chinese Thorawax Root) (Chai Hu) 4%. We note that *Angelica sinensis* also houses endophytic fungi that can have great effects on the metabolome [534]. Xuebijing is an injectable subset of Xue Fu Xhu Yu plus Danshen (*Salviae Miltiorrhizae Radix et Rhizoma*) composed of five Chinese herbs, which are Honghua (*Carthami flos*), Chishao (*Paeoniae radix rubra*), Chuanxiong (*Chuanxiong rhizoma*), Danggui (*Angelicae sinensis radix*), and Danshen (*Salviae miltiorrhizae radix et rhizoma*), particularly used against sepsis [248,257]. A recent study [260] selected hydroxysafflor yellow A (HSYA), vanillin, ligustilide, paeoniflorin, and other substances as the main active ingredients of XueBijing through a comprehensive analysis of metabolomics and network pharmacology. Among them, HSYA showed outstanding performance in promoting endothelial cell proliferation [260].

According to https://sys02.lib.hkbu.edu.hk/cmfid/details.asp?lang=eng&id=F00115 (accessed on 6 May 2025) (and using slightly different names), within the Jun-Chen-Zuo-Shi (Emperor-Minister-Assistant-Courier) principle mentioned above, the components are considered to be: Tao Ren and Hong Hua as Emperor; Chi Shao, ChuanXiong, and Niu Xi as Minister; Sheng Di, Danggui, Jie Geng, Zhi Qiao, and Chai Hu as Assistant; and Gan Cao as Courier.

### Bioactive Molecules in Xue Fu Zhu Yu

We now look at some of the molecules that are considered to be active within the different herbs (Table 4). While this is likely to be far from complete, it shows clearly the known and multiple effects of some of the major bioactive components in the herbs comprising Xue Fu Zhu Yu.

What is also clear is that there is a wealth of literature in some cases and a dearth in others, and comparing subsets of the mixtures leads to an infeasible combinatorial explosion [713]. There are also some consonances, with molecules or classes being common to more than one of the herbs (Table 4), and of course it is well known that natural products can make for successful drugs, even in Western medicine (e.g., [714,715,716,717,718]). It is also of interest that the triterpenoid saponins (here platycodins, saikosaponins, and glycyrrhizin), a class of molecules of increasing importance in natural product drug discovery [719,720,721,722,723,724,725,726,727,728,729,730,731], can also contain most or all of a steroid nucleus [732,733,734,735,736,737] and have good bioavailability [738,739] (probably through their chemical relatedness to steroids and bile acids [740,741,742]). Importantly, such triterpenoids may inhibit toxic amyloidogenesis (e.g., [743,744,745,746,747,748,749,750,751,752,753,754,755,756,757,758,759,760,761,762,763,764,765,766,767,768,769,770,771,772,773,774,775,776,777,778,779,780,781,782]), and indeed appear in other herbal formulae for stimulating blood circulation in blood stasis syndrome (e.g., [783,784,785,786,787]).

To avoid cluttering up Table 4, chemical structures of some of the main components of the herbs in Xue Fu Zhu Yu are given in Table 5.

What is also clear from Table 5 is the large number of different chemical structures of even the main, known bioactive elements in Xue Fu Zhu Yu, as well as their multiple targets (Table 4).

Natural products tend to be larger than purely chemical drugs [788,789,790,791,792,793,794,795,796,797], which may imply that they have even more targets than the average of six mentioned above for pharmaceutical drugs [61]. While relatively little is known of the transporters responsible for their uptake into cells, it is known that pharmaceutical drugs that are taken up do mimic natural products [59].

## 6. Discussion, with a Focus on Mechanism(s) of Action of Xue Fu Zhu Yu Decoction

This article, as a synthetic review [798], has brought together four major themes that had not previously been related to each other:

1. The concept from traditional Eastern medicines of blood stasis, however imperfectly understood mechanistically, as a recognition that in many syndromes blood is not flowing as freely as normal or desirable;
2. The discovery and properties of fibrin amyloid (‘fibrinaloid’) microclots that form when blood clots into an anomalous amyloid form; this can be catalyzed by infectious agents (that were of course unknown to early traditional medicines); as amyloids, as well as by entrapping anti-proteolytic substances, they are resistant to the normal mechanisms of fibrinolysis and can thus block the normal transport of red blood cells (and hence oxygen transport to tissues), leading to many unhealthy sequelae;
3. The recognition that many traditional herbal formulas or cocktails, carefully designed to target multiple things simultaneously and exemplified by Xue Fu Zhu Yu decoction (XFZYD), are efficacious in removing the symptoms (and possibly the causes) of blood stasis;
4. The increases in our understanding of both the molecular constituents of such cocktails, illustrated by Xue Fu Zhu Yu, and some of the molecular targets with which have been found to interact.

For illustrative purposes, these effects include at least the following:

1. **Neuroprotective effects**: XFZYD may exert neuroprotective effects by regulating miRNA expression and promoting synaptic remodeling. A study found that XFZYD could reverse the reduction of BDNF and TrkB in the hippocampus caused by traumatic brain injury and increase the number of synaptic connections, as well as the expression of synaptic-related protein PSD95, axon-related protein GAP43, and neuron-specific protein TUBB3 [519].
2. **Anti-inflammatory effects**: Multiple studies have shown that XFZYD has anti-inflammatory effects. In an alopecia model, XFZYD can significantly inhibit the levels of IL-6, IL-1β, and TNF-α in serum and skin tissue [799].
3. **Promoting angiogenesis**: A study evaluated the angiogenic effect of XFZYD using a PTK787-induced segmental vascular injury zebrafish model. Through various analytical methods, seven active components promoting angiogenesis were identified, including ferulic acid, paeoniflorin, and hesperidin [800].

## 7. Summarizing and Concluding Remarks

The concept of blood stasis is exceptionally important in traditional Asian medicines, and we have here argued that it reflects the fibrinaloid microclots that two of us had discovered. The recognition that herbal formulae such as Xue Fu Zhu Yu are well known (by relevant practitioners) to be of value in treating blood stasis, as well as some of the bioactive molecules that it contains, thus opens up the area of microclots to focused pharmacological analyses. This said, Xue Fu Zhu Yu contains 11 separate herbs, each containing multiple bioactive elements, each of which is likely to have multiple targets (Table 4, and more generally [60,61,801]). Deconvolving the precise effects in different cases is consequently going to be difficult, though progress is being made (Table 4). However, the recognition that microclots may largely equate to or be responsible for ‘blood stasis’ also offers the hope of effective treatments.

Traditional Eastern medicines recognize that, while herbal cocktails such as Xue Fu Zhu Yu are commonly efficacious against blood stasis syndrome (and may be suggested, especially when the individual is unable to visit a suitable practitioner), ideally one should seek to obtain a variant recipe more precisely tailored to the individual’s needs. However, from the perspective of the mechanism(s) of action of the molecules and herbs in XFZYD, future research might focus on the synergistic effects of Xue Fu Zhu Yu components at both the herb and bioactive molecule levels, their pharmacokinetics, their effects on fibrinaloid microclot generation and persistence coupled to any symptom amelioration, and their clinical efficacy in treating chronic diseases, as well as metabolomics methods for their composition and effects. Additionally, the development of standardized biomarkers for BSS and microclot burden will be crucial for personalized treatment strategies.

## Figures and Tables

**Table 2:** Some diseases or syndromes in which fibrinaloid microclots have been observed and which are considered to involve blood stasis.

Disease or Syndrome	Selected References Showing Microclot Formation	Selected References Relating the Disease to Blood Stasis
Alzheimer’s disease	[185,186,187,188]	[80,189,190,191]
(Acute) COVID-19 infection	[192,193,194,195,196]	[197,198,199,200,201,202,203,204]
Diabetes type 2	[119,186,196,205,206]	[73,77,80,207,208,209,210]
Long COVID	[133,134,147,150,211,212,213,214,215]	[200,216,217]
Migraines	[218]	[80,219]
Myalgic encephalopathy/ chronic fatigue syndrome	[220,221]	[217,222,223,224,225] (see also [226])
Parkinson’s disease	[121,186,227,228]	[68,229,230,231]
Rheumatoid arthritis	[211,232,233]	[80,210,234,235]
Sepsis	[236]	[237,238,239,240,241,242,243,244,245,246,247,248,249,250,251,252,253,254,255,256,257,258,259,260,261,262,263]
Septic shock	[236]	[264,265,266,267,268,269,270,271,272,273,274,275,276]
Stroke	[277]	[83,87,278,279,280,281,282,283,284,285,286,287,288,289,290,291,292,293,294,295,296,297,298]

**Table 3:** Diseases involving blood stasis where fibrinaloid microclots are yet to be measured directly. These diseases are mostly vascular or thrombotic. We here ignore classical amyloidoses and cancer.

Disease or Syndrome	Selected References Relating the Disease to Blood Stasis	Comments
Angina pectoris	[80,300,301,302,303,304,305,306,307,308,309,310,311,312,313,314,315,316]	A very obvious example, as vasodilators are the main treatment. The tightening of the chest and shortness of breath are easily explained by microclots blocking capillaries.
Atherosclerosis	[317,318,319,320,321,322,323]	Another very obvious example of fibrinaloid microclots resisting fibrinolysis contributing to atherosclerotic plaques (and later to stroke [277]). The pairing Danshen–Chuanxiong is often used [320,324].
Atrial fibrillation (AF)	[15]	At one level, atrial blood stasis is seen as synonymous as an effect of AF [325,326]. Evidence that fibrinaloid microclots are more a cause than an effect of AF was summarized in [327].
Attention deficit hyperactivity disorder (ADHD)	[328,329,330,331]	Plausibly due to decreased blood flow caused by microclots.
Chronic kidney disease	[79,332,333]	Less likelihood of kidneys excreting microclots if diseased.
Chronic obstructive pulmonary disease (COPD)	[334,335,336,337,338]	Strong hint in the term ‘obstructive’.
Coronary heart disease	[91,92,310,313,339,340,341,342,343,344,345,346,347,348,349,350]	Xue Fu Zhu Yu (a formula to overcome blood stasis) helps [341,343,351,352,353].
Deep vein thrombosis (DVT)	[354,355,356]	TCM classifies DVT as blood stasis in the category of ‘pulse closed’ and ‘femoral swelling’. Xue Fu Zhu Yu helps, and there is evidence [132] that the thromboses are likely to be amyloid in nature.
Dysmenorrhea	[357,358,359,360,361,362]	Note that 17-β-estradiol was one of the first small molecules discovered to induce anomalous clotting [106].
Fibromyalgia syndrome (FMS)	[222,363]	We consider it likely that FMS (and fibrosis [336]) is caused by deposition of fibrin caused by fibrinaloid microclots [141]. There is little actual work on BSS here.
Heart failure and ischemic heart disease	[364,365,366,367]	Various formulas used for this kind of blood stasis.
Metabolic syndrome (MS)	[80,88,368,369]	MS covers a variety of different syndromes; at this stage we do not seek to deconvolve it.
Non-alcoholic fatty liver disease (NAFLD) (since mid-2023 it is called metabolic dysfunction-associated steatotic liver disease (MASLD) [370])	[210,371,372]	
Postural orthostatic tachycardia syndrome (POTS)	[373]	Capillary blocking by fibrinaloid microclots provides a ready explanation for POTS [374] (see also [375]).
Pre-eclampsia (PE)	[376,377,378,379]	PE has a microbial origin [380,381] and is significantly prothrombotic [382].
Pulmonary embolism	[99,383]	
Sub-arachnoid hemorrhage	[384,385]	The only predictor of a later stroke in [386] was ESR (erythrocyte sedimentation rate), a measure of blood stasis.
Thrombotic diseases generally	[387,388,389]	High likelihood of the clots involved being amyloid in nature [132].
Tinnitus	[73,390]	A common accompaniment to diseases (such as long COVID) where microclots are involved and where both can be induced by spike protein (cf. [192] and [391,392,393]).
Transient ischemic attack (TIA)	[289,394,395]	TIA is of course a common precursor to ischemic stroke [396], where amyloid clotting has been demonstrated [277].
Traumatic brain injury (TBI)	[397,398,399,400,401,402,403]	Blood stasis is seen as a core component of (the sequelae of) TBI, which include coagulopathy [404]. Most significantly, Xue Fu Zhu Yu ameliorated neurological deficiencies without impairing blood coagulation in a rat model [401].
Traumatic injury generally	[72,405]	

**Table 4:** Constituent herbs of Xue Fu Zhu Yu, some known bioactive elements it contains, and some known activities.

Herb	Some Known Bioactive Molecules Therein	Some Known Targets or General Properties
Tao Ren; *Semen persicae*; peach kernel (**Emperor**)	Amygdalin [535,536]	Follistatin induction [535]; ERK1/2 activation [536]
Hong Hua; *Flos carthami*; *Carthamus tinctorius* L.; safflower (**Emperor**)	(Hydroxy)safflor yellow A [537,538,539,540,541,542]	Antithrombotic, angiogenic, anticoagulant, antiplatelet. Reviews: [543,544,545,546,547,548]
	Kaempferol, quercetin	Antioxidants/ anti-inflammatory [537]
	Endothelial cell protection	Enhances HIF1-α [537]
Chi Shao; *Radix paeoniae rubra*; *Paeonia lactiflora* Pall; red peony root (**Minister**)	Oxypaeoniflorin	Anti-thrombin [388]
	Paeoniflorin [549]	Anti-stroke [550,551], anti-thrombotic [552,553], anti-inflammatory [554,555,556,557,558], and antioxidant [559]; blocks TGF1β signaling, ERK1/2, JNK1/2, NF-κB, etc. [549,560,561]; deactivation of STAT3 [562]; Akt/Nrf2/GPX4 [231], MAPK [563,564], Raptor [565], TRPV1 [566], HIF-1 α [567], adenosine A_1_ receptor [568,569]. Analgesic [570]. Reviews of nervous system [571] and cardioprotective [572] effects [573]
	Paeonol	Anti-inflammatory, antioxidant, protects endothelium [574]; endothelium-protecting and antiplatelet [575]; antioxidant and anti-inflammatory [576]
*Rhizoma chuanxiong* (*Ligusticum chuanxiong*); Szechaun lovage roots (**Minister**)	Reviews [577,578,579,580]	
	Ligustrazine	Anti-inflammatory [581,582]. Dilates blood vessels, inhibits platelet aggregation, and prevents thrombopoiesis [582]. Anti-anginal [583]. Multiple roles [584]
	Ligustilide (also in Dang Gui and Niu Xi)	Anti-inflammatory and anti-oxidant [585]. Improves lipid metabolism, antioxidant and anti-inflammatory, protects vascular endothelium, inhibits vascular endothelial fibrosis [586]. Senolytic [587]. Cannabinoid receptor 2 activation [588]
	Sekyunolide I (SEI)	Reviews on antioxidant and anti-inflammatory properties [589,590,591]. Other targets of SEI include Nrf2 [592], activity vs NAFLD [593], UVB damage [594], NET formation [595], ischemia-reperfusion injury [596,597,598,599]. It occurs in appreciable concentrations in both ChuanXiong [600,601] and *Angelica sinensis* (Danggui) [602]
	Ferulic acid	Anti-thrombin activity [603]
*Radix achyranthis bidentata* (Niu Xi), also *Cyathulae radix* (**Minister**; may also be a **Courier** [604])	Achyranthine, but no real stand-outs	Seemingly not well understood [605,606]
*Radix rehmanniae* (Di Huang or Sheng Di) (**Assistant**)	Reviews: [607,608,609]	Multiple effects, including anti-inflammation, antioxidation, anti-tumor, immunomodulation, cardiovascular and cerebrovascular regulation [609]. Hypoglycemic [610]
	Iridoid glycosides (such as catalpol and aucuboside)	Catalpol blocks AMPK signaling [611] and promotes angiogenesis [612] and cell migration [613]. Antioxidant via Nrf2/HO-1 [614] and NF-κB [615], Many other references, reviewed in [616,617,618]. Aucuboside is an immunomodulator [619]
	Phenylpropanoid glycosides (such as acteoside)	Acteoside, e.g., stimulates amyloid degradation [620], ameliorates ischemia-reperfusion injury [621], and has many other effects [622], including anticancer effects [623]
*Radix angelicae sinensis* (Chinese angelica) (Dang Gui) (**Assistant**)		
	Z-lingustilide (see above)	
	Ferulic acid	Antioxidant and anti-inflammatory [624]. Nephroprotective [625]. Ameliorates lipid metabolism via the AMPK/ACC and PI3K/AKT pathways [626]
	Sekyunolide I (see above)	
*Radix platycodonis* (Balloonflower root) (Jie Geng) (**Assistant**; may also be a **Courier** [604])	Platycodins [627,628] (triterpenoid saponins)	Many activities [629,630,631], including anti-inflammatory and antioxidant [627,632,633], vasodilatory [634], antiviral [635], antithrombotic [636], autophagy-modulating [637], mitophagy-regulating [638,639]
*Fructus aurantia* (*Citrus aurantium* L.) Bitter orange (Zhi Qiao) (**Assistant**)	Flavones and flavonoids, including sinensetin, tangeretin, 5-demethylnobiletin, and chrysin	Antioxidant, anti-inflammatory [640], and other activities via JAK-STAT3 and PI3K-AKT signaling [641]
*Radix bupleuri* (Chinese Thorawax Root) (Chai Hu) (**Assistant**)	Quercetin [310]	Antioxidant and other properties (some not at all newly discovered [642,643])
	Saikosaponins (triterpenoid saponins) (may involve stimulation by endophytic fungi [644,645,646])	Many activities [647], including antioxidant and anti-inflammatory [471,472,648,649,650,651,652,653,654,655,656,657,658,659,660,661,662,663,664,665,666], as well as anti-fibrotic [667,668,669,670,671,672,673,674,675], anti-HIF-1α [676], antiviral [664,677], anti-sepsis [678,679,680,681]
*Radix glycyrrhizae* (*Glycyrrhiza uralensis* Fisch; Chinese licorice) (Gan Cao) (**Courier**)	Flavanones liquiritigenin and isoliquiritigenin	Anti-oxidant [682], also anti-amyloid [683] and transporter inducers. Reviews [684,685]
	Triterpene saponins including glycyrrhizin, glycyrrhet(in)ic acid	Antiviral and other [666,686,687,688,689,690,691,692,693,694,695,696,697]. Anti-sepsis [698,699,700,701,702,703,704,705,706,707,708,709,710]. Atheroprotective [711,712]

**Table 5:** Chemical structures of some of the constituents in Xue Fu Zhu Yu, as mentioned in Table 4. Most were checked at PubChem in February 2025.

Molecule	Structure
Acteoside	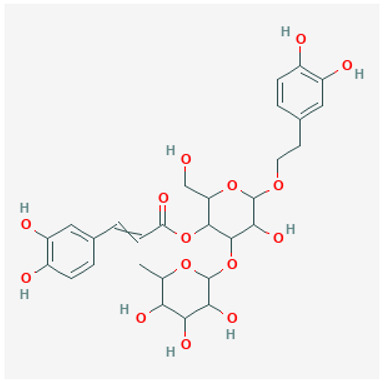
Amygdalin	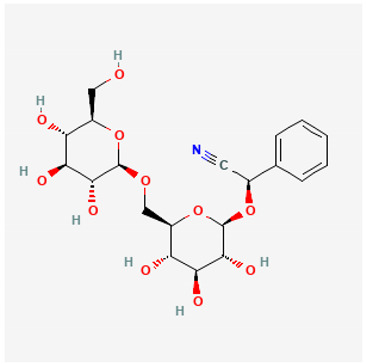
Aucuboside	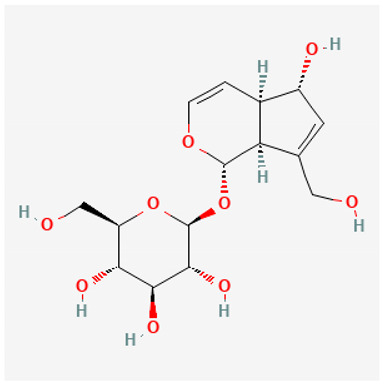
Catalpol	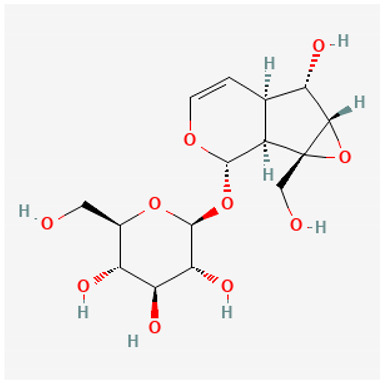
Glycyrrhizin	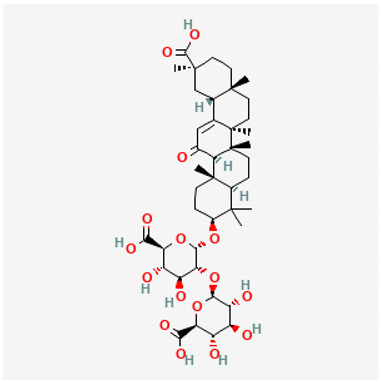
Hydroxysafflor yellow A	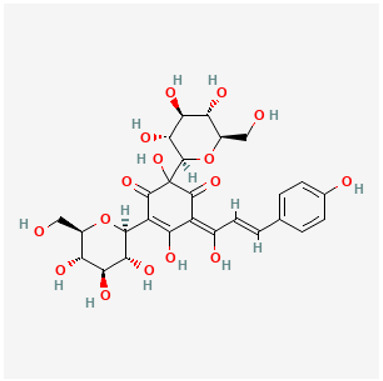
Isoliquiritigenin (2′,4,4′-trihydroxychalcone)	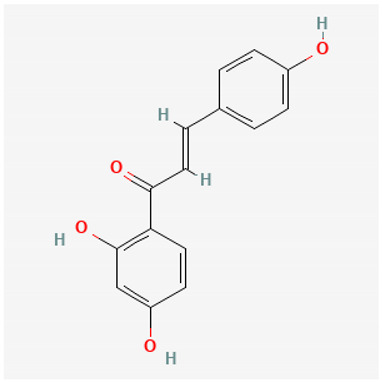
(Z-)Ligustilide	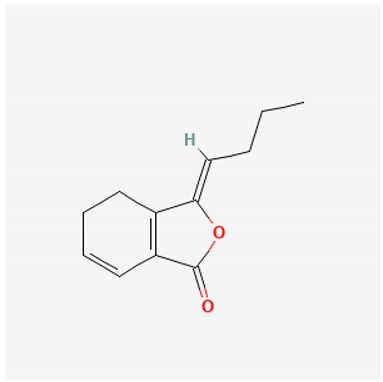
Ligustrazine (2,3,5,6-tetramethylpyrazine)	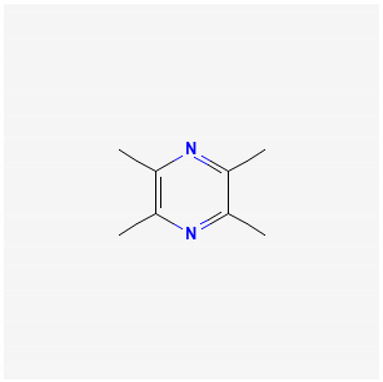
Liquiritigenin 4′, 7-dihydroxyflavanone	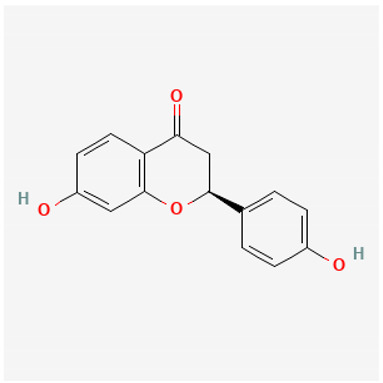
Oxypaeoniflorin	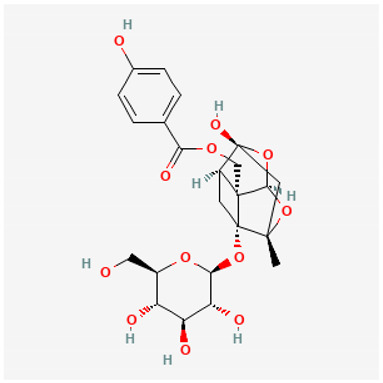
Paeniflorin	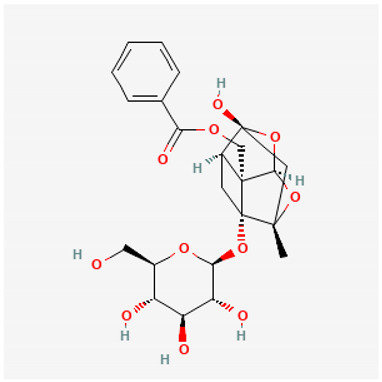
Paeonol	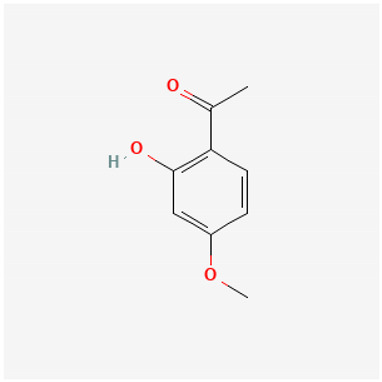
Platycodins: variants of structure at right. One example is below, in which R1 is glucose and R2 is arabinose-rhamnose-xylose-apifuranosyl	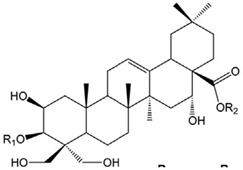
Platycodin D	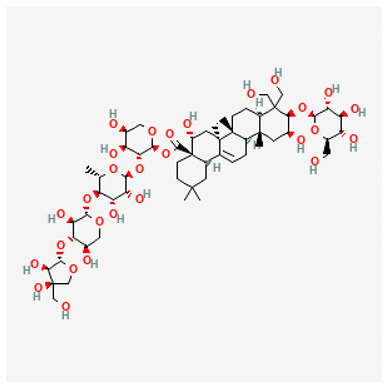
Quercetin	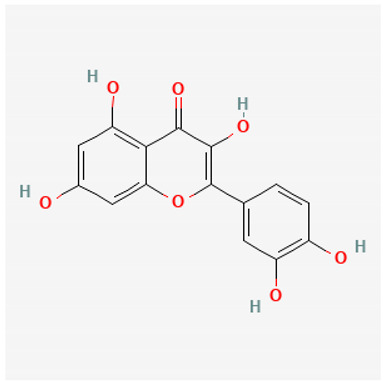
Safflor yellow A	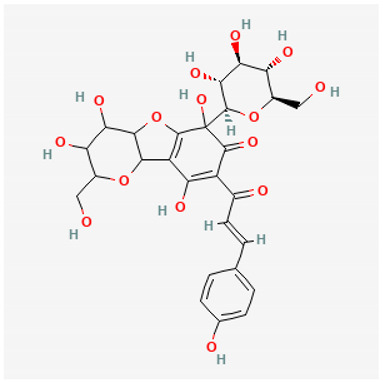
Saikosaponin nucleus	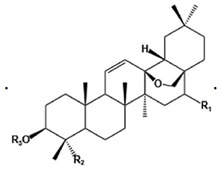
Saikosaponin A	As above, R1 = β-OH, R2 = CH_2_OH
Sekyunolide I	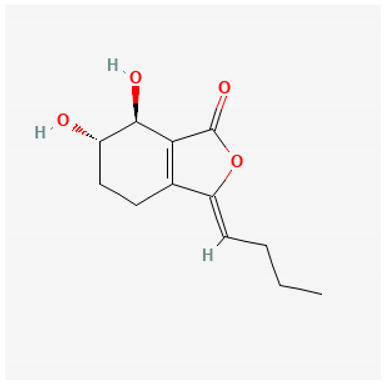

## Data Availability

Not applicable.
